# Cardiac arrest during deep brain stimulation: A case report

**DOI:** 10.1002/ccr3.9147

**Published:** 2024-07-11

**Authors:** Julian Oberholzer, Damien Fayolle, Alberto Vandenbulcke, John G. Gaudet

**Affiliations:** ^1^ Department of Anesthesiology Centre Hospitalier Universitaire Vaudois Lausanne Switzerland; ^2^ Department of Neurology Hôpitaux Universitaires Genève Geneva Switzerland; ^3^ Department of Neurosurgery Centre Hospitalier Universitaire Vaudois Lausanne Switzerland

**Keywords:** deep brain stimulation, parkinson disease, pulmonary arterial hypertension, intraoperative complications, cardiac arrest

## Abstract

We present the case of a 54‐year‐old male with severe Parkinson's disease and chronic, non‐reversible pulmonary artery hypertension who had seizures and a cardiorespiratory arrest during surgery for deep brain stimulation, a minimally invasive procedure usually associated with a low risk of complications. This case illustrates how perioperative changes in antiparkinsonian therapy in patient with multiple comorbidities may significantly affect the risk profile.

## 1 INTRODUCTION

Deep brain stimulation (DBS) is an effective minimally invasive therapeutic strategy for patients with severe movement disorders such as essential tremor, Parkinson's disease, and dystonia. It is also beneficial in patients with refractory psychiatric conditions such as obsessive‐compulsive disorder or Tourette's syndrome.^1^ The procedure involves the stereotactic implantation of electrodes into specific deep brain structures, followed by the connection of subcutaneous wires to a stimulation device usually placed underneath the clavicle. While the implantation of electrodes can be completed under monitored anesthesia care in most cases, tunneling of wires requires general anesthesia with tracheal intubation.

The American Association of Neurological Surgeons (AANS) considers DBS to be a low‐risk procedure.^2^, ^3^, ^4^ Indeed, while the perioperative mortality rate is below 1%, severe adverse surgical events such as intracranial hemorrhage or intracranial abscess are reported in 1%–2% of cases. Cardiopulmonary instability is either very rare or underreported. Here, we present the case of a patient with severe Parkinson's disease and pulmonary artery hypertension who suffered an intraoperative cardiac arrest.

## 2 CASE REPORT

### 2.1 Case history

A 54‐year‐old male patient, diagnosed with severe Parkinson's disease 9 years ago, was scheduled to undergo elective DBS to improve motor symptoms. At the time of admission, he was nearly unable to walk without falling despite maximal dopamine agonist therapy with 1150 mg a day of levodopa combined to opicapone and subcutaneous apomorphine administered by an infusion pump, at a rate of 4 mg/h during the day and 3.5 mg/h at night.

He was classified American Society of Anesthesiologists (ASA) grade 4 due to multiple severe medical problems. Mainly, he was under triple therapy (Tadalafil, Macitentan, Selexipag) for severe pulmonary artery hypertension in the context of untreated sleep apnoea and disseminated lupus erythematosus. Transthoracic echocardiographic examination revealed right ventricular dysfunction with severe chamber dilation and moderate wall hypertrophy. A right heart catheterization measured systolic and mean pulmonary arterial pressures of 107 and 58 mmHg, respectively. Despite being compressed by the right heart, the left ventricle remained functional with an estimated ejection fraction of 65%. Clinically, the patient presented with stable New York Heart Association (NYHA) class 2 dyspnoea.

## 3 METHODS

A multidisciplinary team concluded that the expected benefits of DBS would be superior to the risk associated with such significant comorbidities. Indeed, as the patient required increasingly frequent hospital admissions due to traumatic falls, bilateral subthalamic DBS was thought to be the best option to preserve his ability to stay home.

All antiparkinsonian medications were stopped the night before surgery to facilitate intraoperative neurological testing, except for the continuous subcutaneous apomorphine pump which was stopped the morning of the surgery.^5^, ^6^ Upon arrival from the radiology suite where the subthalamic nuclei were localized relative to the stereotactic frame, the patient appeared restless and anxious. While we aimed to maintain the patient as calm and collaborative as possible during surgery, we also aimed to minimize the cardiorespiratory depressant side effects of anesthetic agents. Our priority was to avoid hypoventilation, as hypoxemia and hypercarbia would further elevate pulmonary arterial pressures. Consequently, we chose to maintain the patient mildly sedated with continuous Dexmedetomidine and small Propofol boluses per need during the initial electrode implantation phase. Prior to surgical start, we placed a right femoral central venous catheter and arterial line to administer vasoactive drugs if necessary.

## 4 RESULTS AND CONCLUSION

Implantation of the first electrode in the right subthalamic nucleus went flawlessly. However, promptly thereafter, the patient lost consciousness and failed to recover after discontinuation of sedation. Within minutes, we observed convulsive movements of his right lower limb that evolved into a generalized tonic–clonic seizure. Following loading doses of Levetiracetam and Lacosamide that successfully stopped the seizure, he had a non‐contrast cerebral CT scan in emergency to exclude surgical complications. In absence of cerebral hemorrhage, decision was made jointly by the neurosurgical, anesthetic, and neurological teams to proceed back to the operating theater after a surveillance period during which the patient remained stable and seizure‐free.^7^ Implantation of the second electrode went well. He was discharged from the recovery room after an uneventful stay.

Soon after his admission in the neurosurgical step‐down unit, his level of consciousness rapidly declined once again and he had multiple refractory episodes of generalized seizure compatible with the diagnosis of status epilepticus. In absence of surgical complications on the second cerebral imaging, he was admitted to the intensive care unit (ICU) where he was intubated to protect the airway and optimize gas exchange. The patient also underwent an MRI which did not show signs of Posterior Reversible Encephalopathy Syndrome (PRESS) or cerebral edema. In this context, invasive intracranial pressure monitoring was not considered a priority. Instead, the reintroduction of subcutaneous apomorphine dramatically accelerated his neurological recovery, suggesting that dopamine agonist withdrawal syndrome (DAWS) may have contributed to this altered state of consciousness. Upon cessation of sedation, he rapidly regained consciousness, was able to respond to command and he was weaned from hemodynamic pharmacological support. Despite persistent mild hypoxemia (PaO2 65 mmHg on 30% FiO2), he remained neurologically and hemodynamically stable for the next 24 h. At this point, the neurosurgical, anesthetic, neurological, and neurocritical care teams jointly cleared him for completion of the surgical procedure (subcutaneous tunneling of wires and implantation of the stimulation device) under full antiparkinsonian therapy, as a step towards further recovery.

We used sevoflurane in combination with remifentanil to induce and maintain general anesthesia.

**FIGURE 1:**
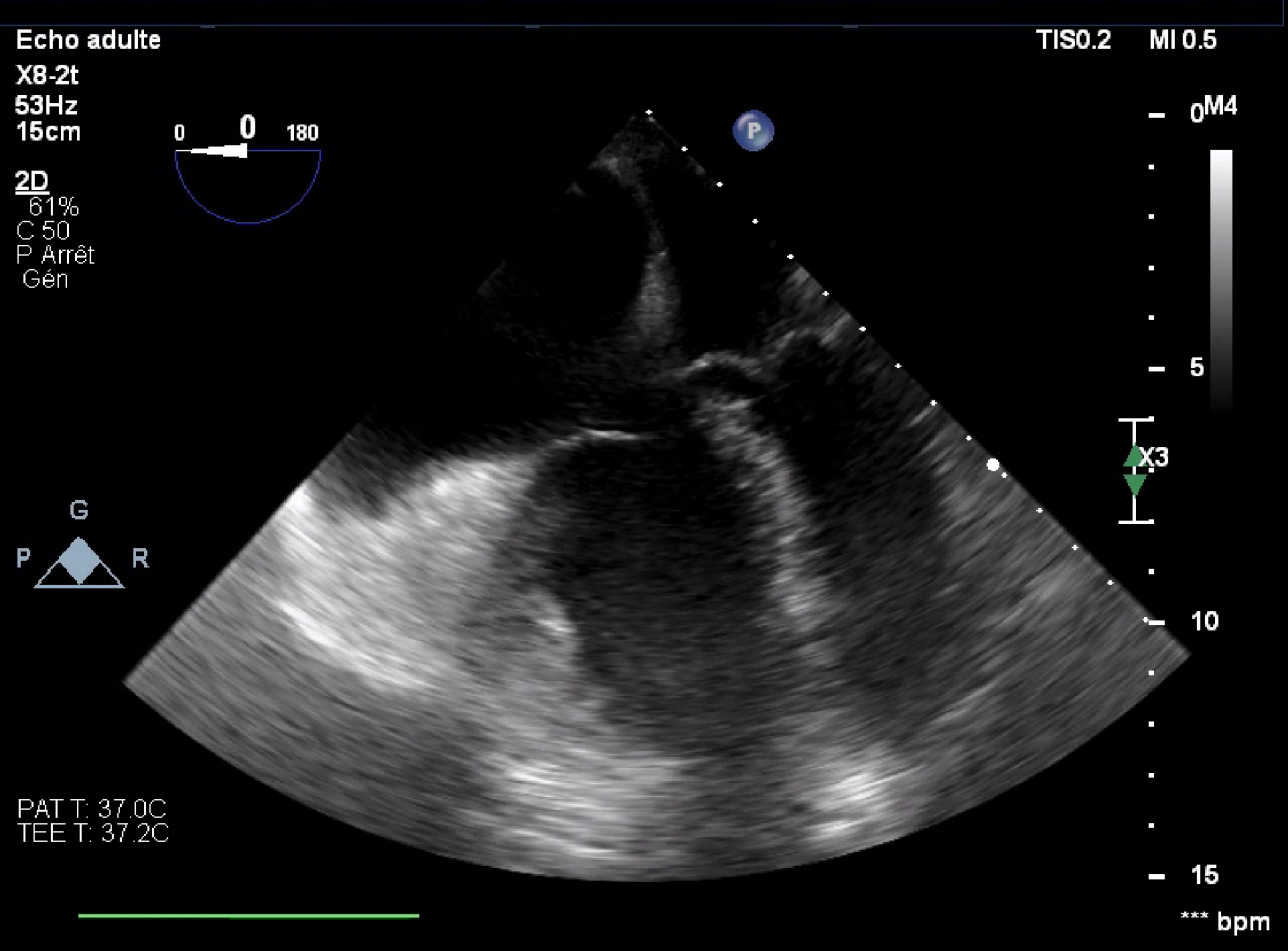
Transesophageal echocardiography after Right ventricle dilatation (ROSC) with hypertrophy of the free wall. Left ventricle is small and hyperdynamic. Pulmonary arterial pressures estimated at 100 mmHg.

Unfortunately, the patient's hemodynamic status worsened despite increasing dobutamine and noradrenaline support, as well as regular adrenaline boluses which contributed to further accelerate his heart rate. The combination of hypoxemia and worsening cardiogenic shock spiraled down until he arrested and required chest compressions for 3 min as the neurosurgical team was closing skin. On return of spontaneous circulation, we excluded the option of using extracorporeal membrane oxygenation (ECMO) given the low probability of weaning the patient off support. On transoesophageal echocardiography, the left ventricle was unable to fill due to right ventricular compression and tachycardia. Pulmonary arterial pressures were estimated at 100 mmHg (Figure 1). In this context, we started an infusion of vasopressin to reduce the positive chronotropic and pulmonary vasoconstrictive effect of adrenaline. Once stabilized, we transferred the patient back to the ICU. There, pulmonary arterial pressures reached a peak of 130 mmHg in the context of left ventricular dysfunction and pneumonia. Aggressive fluid depletion with loop diuretics and antimicrobial therapy improved his hemodynamic status. He was successfully extubated 8 days after surgery and was discharged home on postoperative day 43.

Despite some initial motor improvements and discontinuation of Apomorphine therapy, he continued to experience freezing with falls, requiring further pharmacological adjustments and recurrent hospitalizations. Eventually, Apomorphine was resumed. However, the patient does not exhibit any neurological sequelae from his cardiac arrest.

## 5 DISCUSSION

This case report illustrates two points that are specifically related to the anesthetic management of patients with advanced Parkinson's disease scheduled for DBS.

First, patients with Parkinson's disease are at greater risk of having intraoperative seizures following discontinuation of antiparkinsonian agents, especially when on high‐dose therapy.^8^ Overall, the seizure risk remains relatively low at around 2.5%,^9^ and older age at the time of surgery is associated with a slight risk increase.^10^ DAWS is a well‐described syndrome that initially presents with anxiety and dysphoria and can lead to lethargy if left untreated. While DAWS is not typically associated with seizure activity, dopamine signaling has long been known to affect the seizure threshold.^11^

We propose that the combination of DAWS to common perioperative seizure triggers such as sleep deprivation, fasting, or exposure to bright light may have precipitated seizures. The patient presented in this case report appeared restless and anxious on arrival to the operating theater. He then had several episodes of focal seizures that generalized and became resistant to anticonvulsants. His status rapidly improved after restarting dopamine agonists. Since then, we have revised our perioperative management of antiparkinsonian agents. For DBS, short‐acting drugs such as apomorphine are now typically restarted intraoperatively, at the end of intraoperative neurological testing. For other surgical procedures, apomorphine is continued subcutaneously and other antiparkinsonian drugs are administered enterally through a gastric tube whenever surgery is expected to last longer than 4 h.

Second, patients with advanced Parkinson's disease are at greater risk of pulmonary complications. Indeed, bronchoaspiration pneumonia is approximately four times more likely and remains the leading cause of death in this population.^12^, ^13^ Additionally, patients at all stages of Parkinson's disease show spirometric signs of inspiratory muscles weakness predominantly associated with a restrictive ventilatory dysfunction. Importantly, the restrictive pattern seems to intensify in the “off” state.^14^ It is therefore essential to avoid unintentional over‐sedation, which can impair responsiveness to hypercapnia and hypoxemia. In patients with pulmonary artery hypertension, altered gas exchanges can precipitate right heart failure with catastrophic outcomes, as illustrated by our case report. Under such circumstances, hemodynamic support with adrenaline only can contribute to further elevate pulmonary pressures and thus worsen the situation. Our experience supports the importance of introducing vasopressin as early as possible to support systemic blood pressures without elevating pulmonary blood pressures.^15^

Overall, this case points to the importance of balancing risks and benefits of surgery irrespective of the expected level of tissue damage. Indeed, patients with severe comorbidities have very limited reserve to tolerate the perioperative stress associated with any surgery, including minimally invasive procedures. Whenever the anesthetic risk is significant, it is essential that patients are fully assessed and optimized preoperatively. All options including alternatives to surgery must be considered and discussed by a multidisciplinary team. They must then be clearly presented to the patient.

## AUTHOR CONTRIBUTIONS

**Julian Oberholzer:** Writing – original draft; writing – review and editing. **Damien Fayolle:** Writing – review and editing. **Alberto Vandenbulcke:** Writing – review and editing. **John G. Gaudet:** Supervision; writing – original draft; writing – review and editing.

## FUNDING INFORMATION

The authors have no external funding.

## CONFLICT OF INTEREST STATEMENT

The authors declare no conflicts of interest.

## ETHICS STATEMENT

Not applicable.

## CONSENT

Written informed consent was obtained from the patient to publish this report in accordance with the journal's patient consent policy.

## Data Availability

Data sharing not applicable—no new data generated.
